# Physicochemical Characterization and Antimicrobial Analysis of Vegetal Chitosan Extracted from Distinct Forest Fungi Species

**DOI:** 10.3390/polym15102328

**Published:** 2023-05-16

**Authors:** Iversen Luk Jun Lam, Mariah Aqilah Mohd Affandy, Nasir Md Nur ‘Aqilah, Joseph Merillyn Vonnie, Wen Xia Ling Felicia, Kobun Rovina

**Affiliations:** Faculty of Food Science and Nutrition, Universiti Malaysia Sabah, Kota Kinabalu 88400, Sabah, Malaysia

**Keywords:** chitosan, biodegradable polymer, vegetal, fungi, antimicrobial analysis

## Abstract

The main goal of this investigation is to conduct a thorough analysis of the physical, chemical, and morphological characteristics of chitosan derived from various forest fungi. Additionally, the study aims to determine the effectiveness of this vegetal chitosan as an antimicrobial agent. In this study, *Auricularia auricula-judae*, *Hericium erinaceus*, *Pleurotus ostreatus*, *Tremella fuciformis*, and *Lentinula edodes* were examined. The fungi samples were subjected to a series of rigorous chemical extraction procedures, including demineralization, deproteinization, discoloration, and deacetylation. Subsequently, the chitosan samples were subjected to a comprehensive physicochemical characterization analysis, encompassing Fourier transform infrared spectroscopy (FTIR), scanning electron microscopy (SEM), energy-dispersive X-ray spectroscopy (EDX), X-ray diffraction (XRD), degree of deacetylation determination, ash content determination, moisture content determination, and solubility determination. To evaluate the antimicrobial efficacy of the vegetal chitosan samples, two different sampling parameters were employed, namely human hand and banana, to assess their effectiveness in inhibiting microbial growth. Notably, the percentage of chitin and chitosan varied significantly among the distinct fungal species examined. Moreover, EDX spectroscopy confirmed the extraction of chitosan from *H. erinaceus*, *L. edodes*, *P. ostreatus*, and *T. fuciformis*. The FTIR spectra of all samples revealed a similar absorbance pattern, albeit with varying peak intensities. Furthermore, the XRD patterns for each sample were nearly identical, with the exception of the *A. auricula-judae* sample, which exhibited sharp peaks at ~37° and ~51°, while the crystallinity index of this same sample was approximately 17% lower than the others. The moisture content results indicated that the *L. edodes* sample was the least stable, while the *P. ostreatus* sample was the most stable, in terms of degradation rate. Similarly, the solubility of the samples showed substantial variation among each species, with the *H. erinaceus* sample displaying the highest solubility among the rest. Lastly, the antimicrobial activity of the chitosan solutions exhibited different efficacies in inhibiting microbial growth of skin microflora and microbes found on the peel of *Musa acuminata* × *balbisiana*.

## 1. Introduction

Biopolymers are an attractive class of compounds that possess an array of repeating monomers that are linked through chemical bonding, determined by their distinct chemical structures. These biopolymers are generally categorized into three groups based on their source and synthesis method, namely, natural, microbial, and synthetic biopolymers [1]. Naturally occurring biopolymers, such as proteins, lipids, and polysaccharides, can be extracted from a variety of sources. Meanwhile, the microbiologically synthesized biopolymer, pullulan, is a unique example of a biopolymer that is exclusively synthesized by microorganisms. In contrast, synthetic biopolymers, including polylactic acid (PLA), poly(ε-caprolactone) (PCL), and poly(butylene succinate) (PBS), are commonly employed in modern-day applications [2].

Of particular interest is chitosan, a linear polysaccharide derived from chitin, the second-most abundant polysaccharide found in nature, present in the exoskeletons of crustaceans and insects, as well as in most fungi species [3]. The continuous units of N-acetylglucosamine in chitosan are bonded through β-1,4 glycosidic bonds. Although chitin has a similar structure, it differs from chitosan in that each of its monomers contains an amide group, while chitosan contains an amine group in each of its monomers. Remarkably, the use of chitosan could reduce global food waste and is becoming increasingly popular as a biodegradable coating for food, particularly in the agricultural sector, where it has been shown to effectively preserve food quality [4,5].

Despite the promising potential of chitosan, further research is required to investigate its potential for replacing conventional packaging materials like plastics and paper. To this end, researchers have explored the use of chitosan as a biodegradable film for fruits, with most studies using crustacean-sourced chitosan. However, individuals with a high sensitivity towards crustaceans and their by-products can experience shellfish allergy symptoms due to tropomyosin, the primary allergen. Therefore, vegetal sources of chitosan, such as fungi, may be a suitable alternative, providing similar properties to crustacean chitosan without the associated allergen risks [6].

The current study aims to compare the properties of vegetal chitosan extracted from different species of forest fungi with crustacean chitosan to determine whether there are any notable differences. This investigation has implications for addressing the issue of postharvest losses, a persistent global problem, as chitosan is a promising solution for preserving food quality and reducing waste due to microbial spoilage or quality depletion from transpiration and oxidation. While the use of chitosan in the food industry is not new, there is still much to be explored in terms of its potential for wider-scale application. The results of this investigation could have significant implications for the development of new, sustainable sources of chitosan, as well as for the use of chitosan as an antimicrobial agent in various applications, such as food preservation or medical treatments.

## 2. Materials and Methods

The raw materials used in this study are wood ear fungus or black ear fungus (*Auricularia auricula-judae*), monkey head mushroom or lion’s mane mushroom (*Hericium erinaceus*), oyster mushroom (*Pleurotus ostreatus*), snow fungus (*Tremella fuciformis*), and shiitake mushroom (*Lentinula edodes*). The chitosan powder was purchased from Merck, Germany. The laboratory chemicals and materials used are hydrochloric acids, sodium hydroxide solution, ethanol, ethanoic acid, distilled water, red and blue litmus papers, and plate count agar (PCA) powder.

### 2.1. Extraction of Chitosan

Firstly, the fungi samples were rinsed with tap water to remove any dirt and impurities. They were then oven-dried for 24 h at 60 °C. The dried mushrooms were pulverized with a blender into fine powder. The powdered samples of the fungi were then demineralized with 2 M hydrochloric acid at 60 °C, where the ratio of the powder to the hydrochloric acid was 1:15 (*w*/*v*) while being constantly shaken in a water bath [7]. The insoluble portion was filtered and rinsed until the pH reached neutrality (pH = 7). The residue was then oven-dried at 60 °C for 24 h. Then, the dried powder was treated with 2 M sodium hydroxide solution at 80 °C for the deproteinization process while being constantly shaken in a water bath for 12 h. The ratio of the powder to the sodium hydroxide solution was 1:30 (*w*/*v*) [8]. After that, the mixture was filtered and rinsed until the pH reached 7.0. The residue was then oven-dried at 60 °C for 24 h. After this stage, the chitin was discolored by soaking it in ethanol for 24 h [9,10]. The chitin was then filtered out, rinsed, and dried for 24 h at 60 °C. 

The chitin was then deacetylated for chitosan extraction with 15 M sodium hydroxide solution at 100 °C for 6 h while being constantly shaken in a water bath. The ratio of the chitin powder to the sodium hydroxide solution was 1:15 (*w*/*v*) [9]. The mixture was filtered again and the residue was rinsed with distilled water until the pH reached 7.0. The residue was then oven-dried at 60 °C for 24 h and the chitosan was stored in a desiccator.

### 2.2. Characterisation of Chitosan

The extracted chitosan was characterized by an SEM (Hitachi, SU3800 model, Hitachi City, Japan), an EDX spectroscope (Rigaku, NEX model, Tokyo, Japan), an XRD (Philips, X’pert PRO model, Amsterdam, The Netherlands), and an FTIR spectroscope (PerkinElmer, Spectrum 100 model, Waltham, MA, USA). The surface morphology of the extracted chitosan samples was observed using an SEM. The samples were pretreated and dehydrated to prevent instrumental contamination due to the high vacuum in the SEM (Hitachi, SU3800 model, Japan) [11]. The chitosan samples adhered to a film of carbon, which was then coated with gold and palladium by a sputter coater. The samples were then placed into the magnification chamber to perform the spectroscopy at 15 kV and a magnification of 50,000× [12]. The chitosan samples were mixed with potassium bromide and compressed to form 1 cm diameter tablets. The tablets were then placed into the FTIR (PerkinElmer, Spectrum 100 model, Waltham, MA, USA) spectroscopy to be evaluated for their absorbance in the range of wavenumbers between 600 and 4000 cm^−1^ [8,13,14].

### 2.3. Degree of Deacetylation

The deacetylation degrees of the chitosan were calculated based on the absorbance wavenumbers obtained from the FTIR spectroscopy analysis by utilizing three different absorbance ratios (R = A_1655_/A_2870_, A_1655_/A_3450_, and A_1320_/A_1420_) in a similar equation [15].

Degree of Deacetylation (%) = 100% − (R − 0.03822/0.03133)(1)

### 2.4. X-ray Diffractometry (XRD)

The chitosan samples were placed into an XRD system to run at a voltage of 40 kV. The rate of the scan was set to 2° per minute. The crystallinity index of each sample was then calculated with the formula below. The maximum amplitude of the peak at 20° is denoted as I_110_, whereas the amplitude at 10° is denoted as I_am_ [16,17].

CI_110_ = (I_110_ − I_am_) × 100/I_110_ (2)

### 2.5. Moisture and Ash Content Determination

The chitosan was weighed to a mass of 0.5 g for each sample, and the samples were placed into the moisture analyzer to run the moisture content determination. The obtained values for each sample were recorded in percentages. For the ash content, empty crucibles were weighed and denoted as M_a_. The chitosan was weighed to a mass of 0.1 g for each sample (the masses were denoted as M_b_), placed in a crucible, and burned with an open flame until the samples turned black and stopped emitting white fumes. The crucibles were then placed into a furnace to burn at 550 °C for 12 h. The crucibles were weighed again and denoted as M_c_. The ash content for each sample was calculated using the following formula:

Ash Content (%) = 100 × (M_c_ − M_a_/M_b_) (3)

### 2.6. Solubility Determination

The chitosan was weighed to a mass of 0.1 g for each sample (the masses were denoted as M_a_) and the samples were then mixed into a 30 mL solution of 1% ethanoic acid to dissolve. After stirring for 30 min, the undissolved residues were filtered out and dried in an oven. The residues were then weighed and the values were denoted as M_b_. The solubility of each sample was then computed using the formula shown below.

Solubility (%) = 100 × (M_a_ − M_c_/M_a_) (4)

### 2.7. Antimicrobial Activity

The antimicrobial activity of the chitosan samples was measured by conducting a zone of inhibition test, whereby a small circumference of a PCA was placed with seven drops of each chitosan solution prepared in conducting the solubility determination analysis. However, the agar plates were streaked with cotton buds that were used to collect samples from (i) a human hand and (ii) a banana (*Musa acuminata* × *balbisiana*). The plates were then incubated at 37 °C for seven days in an incubator. The plates were then observed to check whether or not there is any microbial growth within the inhibition zone [18].

### 2.8. Statistical Analysis

The obtained quantitative data were analyzed using the 28th version of the IBM Statistical Package for Social Sciences (SPSS) software. The data were first analyzed with a one-way ANOVA test and, if the ANOVA tests showed a significant difference, Tukey’s honest significant difference test was used to analyze the differences between the means. The tests were carried out at a 95% significance level (*p* ≤ 0.05).

## 3. Results and Discussion

### 3.1. Yield of Chitin and Chitosan

Through the intricate processes of chitin extraction, each species of fungus yielded a product with similar characteristics, though they differed in color and viscosity in their wet state. A comparative analysis of Table 1 reveals that each species also yielded varying amounts of chitin. *T. fuciformis* fungus produced the lowest amount of chitin (2.74% ± 0.78), whereas *A. auricula-judae* fungus produced the highest amount of chitin (55.97% ± 3.62) as compared with the other species from an initial sample weight of 50 g. The wet products from demineralisation exhibited higher viscosity and were gelatinous in *A. auricula-judae* and *L. edodes*. Chemical interactions between the polysaccharides in the fungi and the hydrochloric acid produced hydrocolloids, which were formed from the strands of polysaccharides that absorbed and entrapped water, thus increasing the viscosity or gelatinous character in the wet state of the demineralised samples, as reported by Tu et al. [19]. These gelatinous samples had to be mechanically broken down into smaller fragments to enable processing of a larger surface area in the deproteinization stage, where the calcium carbonate and calcium phosphate within the tissues of the fungal cells were degraded. Doing so allowed easier penetration of the sodium hydroxide molecules to deproteinize and deacetylate the samples so that more purified chitosan could be produced [20]. The samples displayed a finer and less viscous texture, similar to wet sand, after being deproteinized in a 2 M NaOH solution, and the reduction in viscosity was contributed by the hydrolytic potential of sodium hydroxide, which broke down the protein and polysaccharide molecules responsible for the gel-like structures formed from the hydrocolloids of the demineralised products [19]. The discolouration process showed no significant observable changes in color, but the product immediately formed globular particles when added to the ethanol solution in terms of shape and texture. This may be attributed to the ineffectiveness of the discolouration process, as there was a possibility that a layer of hardened protein residue may have formed on the dehydrated product. The protein residue may be denatured by the ethanol and formed barrier-like structures on the chitin, preventing the penetration of the ethanol to decolourise the natural pigments in the chitin [21].

After the deacetylation process, which was used to form chitosan, there was a significant reduction in the viscosity of the products in their wet state. This may be attributed to the hydrolytic action contributed by the strong sodium hydroxide solution used as the deacetylating agent. The resistant polysaccharide macromolecules that were unhydrolysed during the deproteinization stage were probably broken down during the deacetylation stage because a higher molarity of sodium hydroxide solution was used (15 M). Thus, the molecular weight of the samples may have been reduced because of the hydrolysis of the polysaccharide macromolecules within the matrices of the samples, leading to a more rigid form [22]. In terms of the yield of the samples, *A. auricula-judae* fungus also yielded the highest amount of chitosan (15.67% ± 1.90) as compared with the rest, which is shown in Table 1. On the other hand, *T. fuciformis* fungus yielded the lowest among the other species of fungi. Despite its popularity in the scientific community for its valuable polysaccharides with potent anti-oxidative and anti-ageing characteristics [23], there are no publications mentioning its use as a potential source for the compounds.

When compared with different sources of chitosan that were previously published, fungal sources of chitosan were much lower than the rest, especially crustacean sources, which were between 14 and 45% [9]. Chitosan is higher in crustacean sources because most of the structural integrity of crustacean shells is supported by chitin itself and calcium compounds such as calcium carbonate and calcium phosphates, whereas in fungal sources, chitosan is found in smaller amounts because of the bigger structural support provided by the polysaccharides and proteins within the tissues of the fungi. Chitin is mostly available in the striped parts, which are the structurally more rigid parts of a fungus [24].

### 3.2. Characterisation of the Vegetal Chitosan

#### 3.2.1. Scanning Electron Microscopy (SEM)

**Figure 1:**
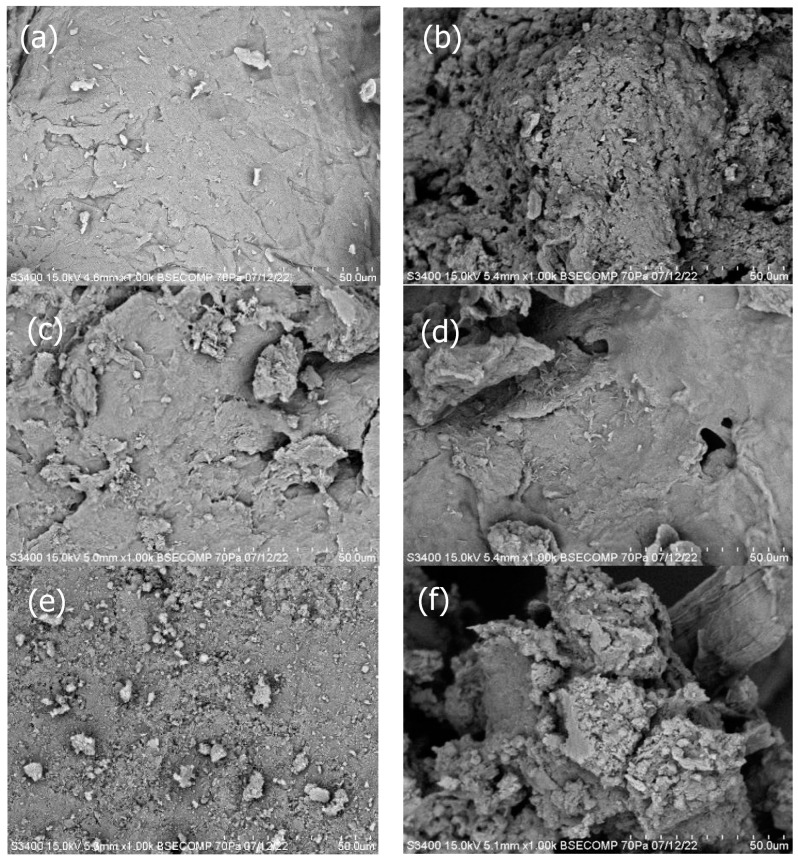
SEM micrographs of the (**a**) crab chitosan powder (1000×), (**b**) *H. erinaceus* (1000×), (**c**) *L. edodes* (1000×), (**d**) *P. ostreatus* (1000×), (**e**) *A. auricula-judae* (1000×), and (**f**) *T. fuciformis* (1000×) samples.

With reference to Figure 1, it can be discerned that the samples of crab chitosan, *L. edodes*, *P. ostreatus*, and *A. auricula-judae* portray a strikingly membranous surface, replete with fibril-like structures that lend a remarkable texture to their appearance. Additionally, the surface also showcases a smattering of uneven-sized particles that bear an uncanny resemblance to the pioneering discoveries of Nikolova et al. [25]. Conversely, upon close inspection of the samples of *H. erinaceus* and *T. fuciformis*, a markedly porous and fibrous surface becomes manifest, featuring clusters of agglomerated particles that are evocative of the seminal works of Oh et al. [26].

#### 3.2.2. Energy Dispersive X-ray (EDX) Spectroscopy

**Figure 2:**
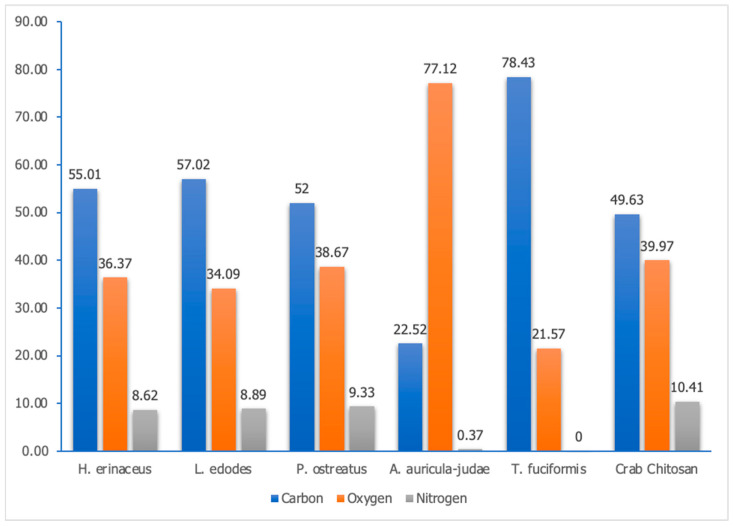
The weight in percentage of carbon, oxygen, and nitrogen in the *H. erinaceus*, *L. edodes*, *P. ostreatus*, *A. auricula-judae*, *T. fuciformis*, and crab chitosan powder samples obtained through EDX spectroscopy.

By virtue of an EDX spectroscopy, the crucial trinity of elements, namely, carbon, oxygen, and nitrogen, were scrutinized in the samples portrayed in Figure 2. The percentage weight of said elements was then juxtaposed against the values obtained from the crab chitosan sample, which served as the control. Remarkably, the majority of the samples, namely, *H. erinaceus*, *L. edodes*, *P. ostreatus*, and *T. fuciformis*, yielded results tantamount to that of crab chitosan, thereby lending credence to the notion that the extracted samples are, indeed, chitosan. However, the A. auricula-judae sample evinced a composition that was starkly incongruous with the chemical signature of chitosan.

As expounded upon by Wolfgong [27], it is conceivable that the X-rays emitted during the EDX spectroscopy might have been spurious, a product of the detection system being configured in a manner that precipitated an erroneous reading. It is plausible, therefore, that the porous point of X-ray emission could have contributed to the aforementioned aberration in the results obtained in this study. Furthermore, it is noteworthy that the scant proportion of nitrogen discernible in the samples of *A. auricula-judae* and *T. fuciformis* could be ascribed to the fact that most detector designs feature a feeble detection system for nitrogen, rendering them somewhat unreliable when estimating the quantity of the said element in a wide array of materials.

#### 3.2.3. Fourier Transform Infrared Spectroscopy (FTIR)

**Figure 3:**
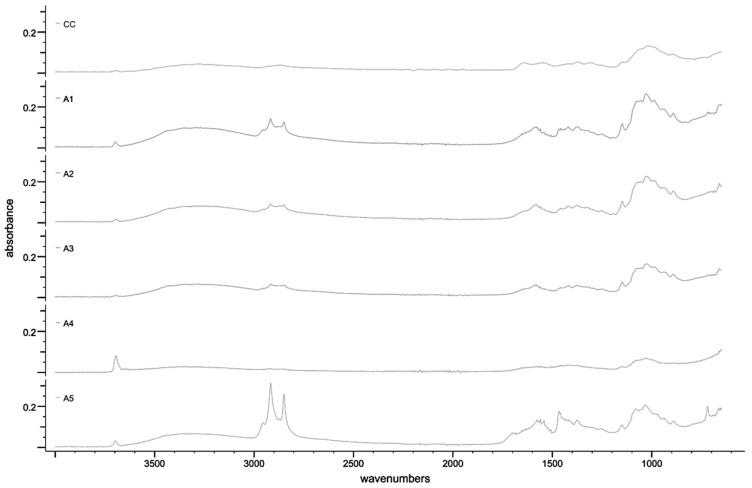
FTIR spectra for the (A5) *T. fuciformis*, (A4) *A. auricula-judae*, (A3) *L. edodes*, (A2) *H. erinaceus*, (A1) *P. ostreatus*, and (CC) crab chitosan powder samples.

With a view to analyzing the FTIR spectra of the quintet of extracted chitosan samples, a comparison was drawn on the spectrum obtained from the analysis of chitosan powder derived from crabs, as showcased in Figure 3. It was observed that, within the realm of single bonds (2500–4000 cm^−1^), all spectra evinced an absorption ranging from 3692 to 3699 cm^−1^, which in turn signified the presence of water or hydroxyls. Additionally, significant absorbances were also detected within the range of 3268–3327 cm^−1^, indicative of the stretching of O-H bonds and, by extension, the existence of hydroxyl groups [28].

Of particular note are the broad and intense peaks of the absorptions discernible in the *P. ostreatus*, *H. erinaceus*, *L. edodes*, and *T. fuciformis* samples within the aforementioned range, which may be suggestive of the involvement of the O-H bonds in inter- and intramolecular hydrogen bonding with water and other water-soluble compounds [29]. Notably, the samples within the range of 2847–2955 cm^−1^ display absorbances that suggest the stretching of C-H bonds, which are primarily found in lipids. Ergo, it stands to reason that there were unhydrolyzed lipid molecules present within the samples [28]. In this context, it is pertinent to mention that, while the *T. fuciformis* sample exhibited the highest absorbance in this regard, the *A. auricula-judae* sample displayed the lowest, thereby providing insight into the concentration of lipids in each sample.

Regarding the double bond region (1500–2000 cm^−1^), all samples exhibited a similar absorbance pattern, but with varying intensities within the range of 1559–1703 cm^−1^. According to Nandiyanto et al. [28], this region showed a stretch between the C=N and C=O in the regions of the amide I and II bands, along with a bend in the N-H bonds, indicating the presence of primary and secondary amides. The *T. fuciformis* sample demonstrated the highest intensity of the stretch in the amide bands, whereas the *A. auricula-judae* sample showed the lowest, implying a high efficiency of the deacetylation process for converting chitin into chitosan.

Considering the chemical structure of chitin and chitosan, chitin contains acetylated amide groups, which are relatively scarce or negligible in chitosan. The amides in chitin undergo deacetylation to form amines in the chitosan structure. It may be assumed that chitosan produced from the *T. fuciformis* sample had the lowest degree of deacetylation. However, the intensities of specific wavenumbers alone may not provide a reliable indicator of chitosan’s purity or degree of deacetylation.

Within the fingerprint region (600–1500 cm^−1^), the range between 1147 and 1160 cm^−1^ for all samples demonstrated sufficient absorbance intensity, indicating the stretching of an asymmetric C–O bond and potentially signifying the presence of a glycosidic linkage within the samples [29]. Based on the wavenumbers alone, it may be concluded that all the extracted samples are, indeed, chitosan.

#### 3.2.4. Degree of Deacetylation

**Figure 4:**
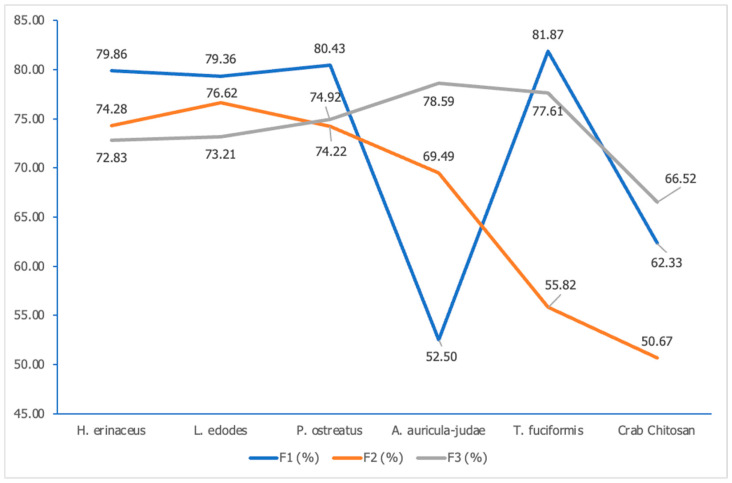
Degrees of deacetylation computed using three different absorbance ratios (F1—A_1655_/A_2870_, F2—A_1655_/A_3450_, F3—A_1320_/A_1420_) for the *H. erinaceus, L. edodes, P. ostreatus*, *A. auricula-judae*, *T. fuciformis*, and crab chitosan powder samples.

The deacetylation degree of chitosan reflects mostly its physicochemical properties and, subsequently, its antimicrobial and antioxidative capabilities [30]. Through computations with different absorbance ratios, the deacetylation degrees of the samples were different based on each ratio used; Figure 4. When the A_1655_/A_2870_ ratio was used, most of the samples (*H. erinaceus*, *L. edodes*, *P. ostreatus*, and *T. fuciformis*) showed the highest deacetylation degree (79.86%, 79.36%, 80.43%, and 81.87%, respectively) as compared with values were obtained through different absorbance ratios. However, according to Kasaai [29], the use of the A_1655_/A_2870_ ratio produced inaccurate results, especially when the sample was mostly acetylated. This might mean that the values obtained using this ratio could possibly have been higher or lower than the actual values.

Through the use of the A_1655_/A_3450_ ratio, the majority of the samples comprising the *P. ostreatus*, *T. fuciformis*, and crab chitosan samples displayed the lowest degrees of deacetylation when compared with its counterparts in the use of the other two absorbance ratios. The deduction based on the 1655 cm^−1^ and 3450 cm^−1^ bands was said to be unsuitable or, at the very least, had some limitations. This is because the water content is a potent factor in the increment of the absorbance intensities as water may cause interference to the bands by causing them to stretch, especially at the amide band and O–H bonds in the 1655 cm^−1^ and 3450 cm^−1^ bands, respectively [29].

For the final ratio used (A_1320_/A_1420_), the degrees of deacetylation obtained are not significantly different (*p* ≤ 0.05) from the values achieved using the other two ratios. In terms of precision and accuracy, the A_1320_/A_1420_ ratio was thought to be the best to be used because the absorbance intensities of the 1320 cm^−1^ and 1420 cm^−1^ bands are not affected by the relative humidity and the intra and intermolecular hydrogen bonds. It was also stated that the degrees of acetylation obtained using the ratio were similar to the deacetylation degrees determined by ^1^H and ^13^C NMR spectroscopy [29].

Theoretically, highly purified chitosan with good solubility is linked to a high deacetylation degree, which is around 85–95% [31]. However, the industrially produced crab chitosan powder displays a range between 51 and 67% for its deacetylation degree, which is considered a low deacetylated degree of chitosan because the minimum requirement to be classified as chitosan is a deacetylation degree of at least 50%. Thus, it can be said that chitosan with a deacetylation degree of less than 100% is a mixture of chitin and chitosan [32,33]. The reason the crab chitosan powder displayed an average deacetylation degree might be the possible degradation that frequently occurs in highly deacetylated chitosan, because the crab chitosan powder has been kept for quite some time [34].

#### 3.2.5. X-ray Diffractometry (XRD)

The XRD results of the chitosan samples reveal remarkably sharp peaks at 20°, a clear indication that the extracted chitosan samples possess a crystalline structure. Meanwhile, the peaks at 10°, except for the crab chitosan sample, exhibit a broader and less intense amplitude that reflects the feeble amorphous structure of the β-chitin chains present in the chitosan. The sparse distribution of the β-chitin chains contributes to the augmented crystallinity of the chitosan. Nevertheless, the XRD pattern of the *A. auricula-judae* sample exhibits conspicuous peaks at around 37° and 51°, signifying the existence of other elements within the chitosan that have contributed to the overall compound’s crystallinity [16,17].

**Figure 5:**
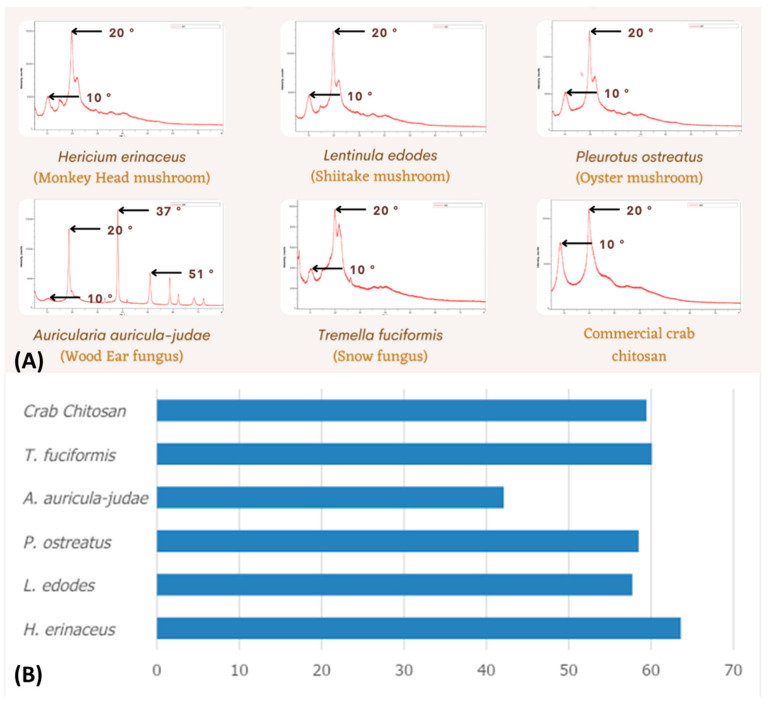
(**A**) XRD patterns and (**B**) crystallinity indices for the crab chitosan powder, *Hericium erinaceus*, *Lentinula edodes*, *Pleurotus ostreatus*, *Auricularia auricula-judae*, and *Tremella fuciformis* samples.

As depicted in Figure 5, the crystallinity indices of chitosan samples indicate that the *H. erinaceus* sample exhibits the highest crystallinity index (63.61%), whereas the *A. auricula-judae* sample demonstrates the lowest (42.11%). The crab chitosan’s crystallinity index was recorded at 59.44%. The remaining samples did not deviate significantly from the control sample concerning crystallinity, thus buttressing the characteristic features of chitosan.

#### 3.2.6. Moisture and Ash Content Determination

Table 2 lays bare the results of the ash content analysis conducted on each of the chitosan samples. While the crab chitosan sample displayed the least amount of ash, at a mere 0.597% ± 0.26, the *L. edodes* sample generated the highest ash content, at an impressive 8.140% ± 0.59. This disparity in ash content of the extracted samples versus the crab chitosan provides compelling grounds to infer that the demineralisation of the fungi samples was carried out effectively and to completion. Moreover, the β-glucans present in the fungi samples may have safeguarded some of the residual minerals from demineralisation during the initial stage of extraction [24].

Notably, the *L. edodes* sample ranks as the sample with the highest moisture content, while the *P. ostreatus* sample ranks lowest in this regard. Commercial chitosan, as per Ghannam et al. [35], typically bears less than 10% of moisture, notwithstanding chitosan’s hygroscopic nature. Therefore, the chitosan extracted from the *L. edodes* sample must be viewed as the most vulnerable to storage-related degradation, as heightened moisture in chitosan triggers the compound’s breakdown, eventually culminating in a decline in its deacetylation degree [36].

#### 3.2.7. Solubility Determination

**Figure 6:**
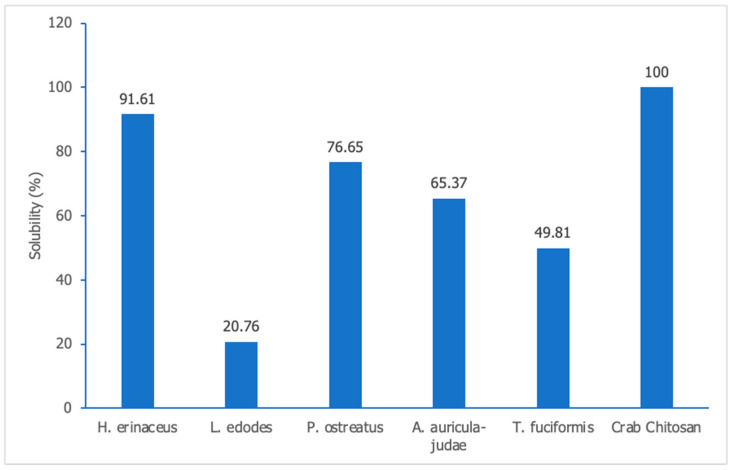
The solubility of the extracted chitosan samples in 1% ethanoic acid.

The solubility of the chitosan samples is showcased graphically in Figure 6. Notably, the *H. erinaceus* sample outperformed its counterparts with its impressive solubility, whereas the *L. edodes* sample languished with the lowest solubility. The solubility of chitosan is a perplexing phenomenon that evades simplistic explanation, as evidenced by prior scholarly works. Aranaz et al. [37] propounded that chitosan’s solubility is subject to multiple factors such as pH, temperature, molecular weight, and other enigmatic parameters beyond the purview of this study. Therefore, the meager solubility of the *L. edodes* sample could be attributed to any one of these factors or a combination of them working in unison to restrict its solubility.

### 3.3. Antimicrobial Activity

The results of the antimicrobial analysis are tabulated below (Table 3). It can be seen that most hand sampling plates are free from microbial growth within the inhibition zone. However, the control (distilled water) for the hand sampling displayed a similar outcome as well. This may infer that the microbes present on the hand during sampling were not significant. For the banana sampling, the control showed a populated growth of microbes. Most of the chitosan samples were not able to strongly inhibit the growth of microbes within the inhibition zone, except for the *H. erinaceus* sample. This proves that a higher solubility is related to increased antimicrobial activity [38].

## 4. Conclusions

In summation, the physicochemical and morphological attributes of the extracted chitosan samples were successfully characterized. It is noteworthy to mention that each fungal species yielded different percentages of chitin and chitosan; however, the color of the chitosan produced was not as pristine as previously reported in the literature. The SEM analysis of the surface morphology was deemed successful as it aligned with findings in various publications. The EDX spectroscopy results confirmed the extraction of chitosan in certain samples (*H. erinaceus*, *L. edodes*, *P. ostreatus*, and *T. fuciformis*), while the FTIR spectroscopy displayed similar absorbance patterns for all samples, with differing peak intensities according to species. Moreover, the XRD patterns for all samples were nearly identical, except for the *A. auricula-judae* sample, which showed a sharp Bragg’s peak at ~37° and ~51°. Notably, the crystallinity index for this sample was lower by around 17% compared with the other samples. The *T. fuciformis* sample had the closest ash content to the crab chitosan, indicating that it is the least stable, while the crab chitosan is the most stable among the samples in terms of degradation rate. The *H. erinaceus* sample demonstrated the highest solubility, which was also the closest to the 100% solubility of the crab chitosan. Lastly, the antimicrobial activity of the chitosan solutions revealed varying efficacies in inhibiting the microbial growth of skin microflora and microbes found on the peel of *Musa acuminata × balbisiana*. It can be inferred that the solubility of the chitosan plays a pivotal role in determining the antimicrobial effectiveness of the chitosan, as higher solubility could result in a greater dispersion and dissolution of the chitosan molecules within a solution or a matrix.

## Figures and Tables

**Table 1:** The chitin and chitosan yield of each species of fungi (dry weight).

Type of Fungus	Chitin Yield (%)	Chitosan Yield (%)
*H. erinaceus*	17.13 ± 1.33 ^c^	2.69 ± 0.22 ^bc^
*L. edodes*	35.17 ± 4.25 ^b^	4.28 ± 0.35 ^b^
*P. ostreatus*	6.38 ± 0.64 ^d^	4.08 ± 0.39 ^b^
*A. auricula-judae*	55.97 ± 3.62 ^a^	15.67 ± 1.90 ^a^
*T. fuciformis*	2.74 ± 0.78 ^d^	2.74 ± 0.78 ^d^

^a–d^—Any two values of mean ± standard deviation (n = 3) with the same letter are not significantly different (*p* ≤ 0.05) from each other.

**Table 2:** The moisture content and ash content of each species of fungi.

Type of Fungus	Moisture Content (%)	Ash Content (%)
*H. erinaceus*	3.520 ± 0.24 ^ab^	7.817 ± 0.24 ^d^
*L. edodes*	8.443 ± 0.59 ^c^	8.140 ± 0.59 ^d^
*P. ostreatus*	2.803 ± 0.61 ^a^	5.690 ± 0.61 ^c^
*A. auricula-judae*	3.440 ± 0.35 ^ab^	6.743 ± 0.35 ^cd^
*T. fuciformis*	2.910 ± 0.86 ^a^	2.997 ± 0.86 ^b^
Crab Chitosan	5.013 ± 0.26 ^b^	0.597 ± 0.26 ^a^

^a–d^—Any two values of mean ± standard deviation (n = 3) with the same letter are not significantly different (*p* ≤ 0.05) from each other.

**Table 3:** The results of antimicrobial analysis of the extracted chitosan samples.

Type of Fungus	Hand Sampling	Banana Sampling
*H. erinaceus*	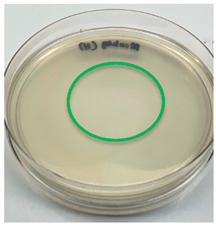	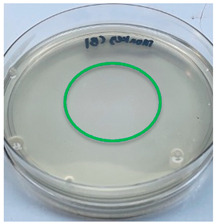
*L. edodes*	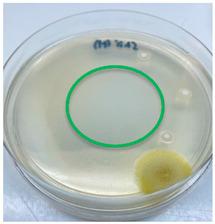	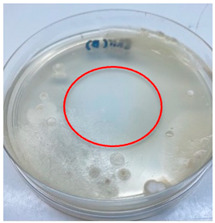
*P. ostreatus*	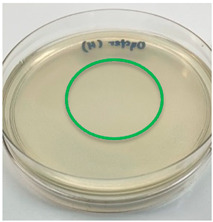	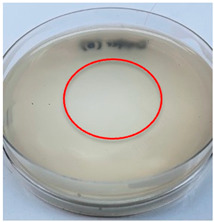
*A. auricula-judae*	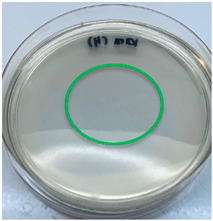	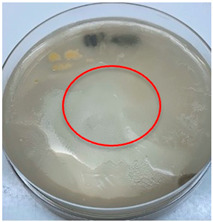
*T. fuciformis*	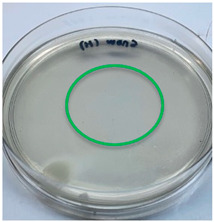	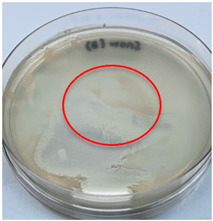
Crab chitosan	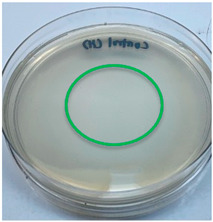	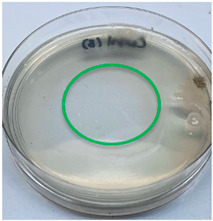
Control (distilled water)	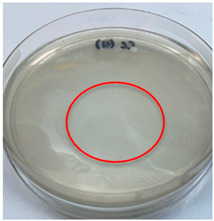	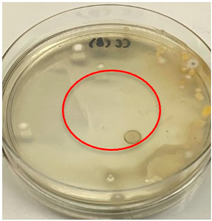

A green marking indicates there is no growth within the inhibition zone (positive inhibition), whereas a red marking indicates otherwise (negative inhibition).

## Data Availability

Not applicable.
